# An unusual cause of granulomatous disease

**DOI:** 10.1186/1472-6890-7-1

**Published:** 2007-03-02

**Authors:** Andrew PC Mclean-Tooke, Catherine Aldridge, Kimberley Gilmour, Bernard Higgins, Mark Hudson, Gavin P Spickett

**Affiliations:** 1 Department of Immunology, Royal Victoria Infirmary, Newcastle-Upon-Tyne, UK; 2 Department of Microbiology, Royal Victoria Infirmary, Newcastle-Upon-Tyne, UK; 3 Molecular Immunology, Great Ormond Street Hospital, London, UK; 4 Department of Respiratory Medicine, Freeman Hospital, Newcastle-Upon-Tyne, UK; 5 Regional Liver Unit, Freeman Hospital, Newcastle-Upon-Tyne, UK

## Abstract

**Background:**

Chronic granulomatous disease (CGD) is an inherited disorder of phagocytic cells caused by an inability to generate active microbicidal oxygen species required kill certain types of fungi and bacteria. This leads to recurrent life-threatening bacterial and fungal infections with tissue granuloma formation.

**Case presentation:**

We describe a case of X-linked Chronic granulomatous disease (CGD) diagnosed in an 18-year-old male. He initially presented with granulomatous disease mimicking sarcoidosis and was treated with corticosteroids. He subsequently developed Burkholderia cepacia complex pneumonia and further investigation confirmed a diagnosis of CGD.

**Conclusion:**

Milder phenotypes of CGD are now being recognised. CGD should be considered in patients of any age with granulomatous diseases, especially if there is a history of recurrent or atypical infection.

## Background

Chronic granulomatous disease (CGD) is an inherited disorder of phagocytic cells resulting in an inability of phagocytes to undergo the respiratory burst required kill certain types of fungi and bacteria. This leads to recurrent life-threatening bacterial and fungal infections as well as persistent tissue granuloma formation. It was initially described in the 1950s as a syndrome of recurrent infections, hypergammaglobulinemia, hepatosplenomegaly, and lymphadenopathy in boys who invariably died in the first decade of life [1]. The majority of cases are detected before the age of five, with 90% detected before the age of fifteen with the diagnosis usually considered due to a history of recurrent, unusual or persistent infections. CGD is caused by mutations in components of the NADPH oxidase system. Identification of this defect has allowed milder phenotypes to be identified which may be associated with milder disease or atypical features resulting in subsequent delay in presentation and diagnosis until adulthood [2-4].

## Case presentation

A 21-year-old gentleman initially was referred with weight loss, fevers and hepatosplenomegaly. He had previously been investigated for lymphadenopathy. A left inguinal lymph node excision 3 years earlier had shown "unusual histology, possibly reactive or due to a low-grade lymphoma" and a decision for close follow up was made. A second lymph node biopsy was performed 1 year later for persistent right cervical and right inguinal lymphadenopathy. Histology showed granulomatous lymphadenitis, again with unusual histology, reported as "possibly due to sarcoid or reactive secondary to infection". At this time he was also noted to have hepatosplenomegaly, abnormal liver function and pancytopenia. A liver biopsy was performed prior to referral showing abnormal architecture, lymphoid proliferation and multiple non-caseating epitheliod granulomas.

On examination he was pale with low-grade pyrexia of 37.5°C. Cardiovascular and respiratory examinations were normal, but he had cervical lymphadenopathy. Abdominal examination revealed massive hepatosplenomegaly without ascites. Computed Tomography of the chest and abdomen showed hepatosplenomegaly with mediastinal and para-aortic lymphadenopathy suspicious of lymphoma. Pulmonary function testing showed decrease in diffusion capacity. Liver function and full blood count were abnormal (Table 1). Serum ACE (angiotensin converting enzyme) was raised at 80 U/L (normal range 8–52). Other bloods including liver screen, autoimmune screen and virology screen were all negative.

A clinical diagnosis of sarcoidosis was made and he was commenced on steroid treatment with prednisolone 40 mg daily. Within two days his pyrexia settled, his liver function improved and his splenomegaly improved. He was discharged home on 20 mg prednisolone. Over the next twenty-four months, his liver function continued to improve and the patient remained clinically well. However, his pancytopenia worsened and a bone marrow biopsy revealed normal haematopoesis with a few non-caseating granulomas. The pancytopenia was felt to be due to hypersplenism and the decision was made for him to undergo splenectomy.

During admission for splenectomy the patient complained of cough and was noted to be pyrexial and tachycardic. A chest radiograph revealed left basal consolidation with right basal changes. Despite a two-week course of moxifloxacin he remained symptomatic with cough, night sweats, fevers up to 40°C and was noted to be hypoxic. Computed Tomography of the thorax showed mediastinal lymphadenopathy with extensive consolidation, and a lung biopsy showed non-specific inflammation. Microbiological culture of the biopsy specimen grew a bacteria belonging to the *Burkholderia cepacia complex*, *Burkholderia multivorans *which was subsequently confirmed on broncho-alveolar lavage.

**Figure 1:**
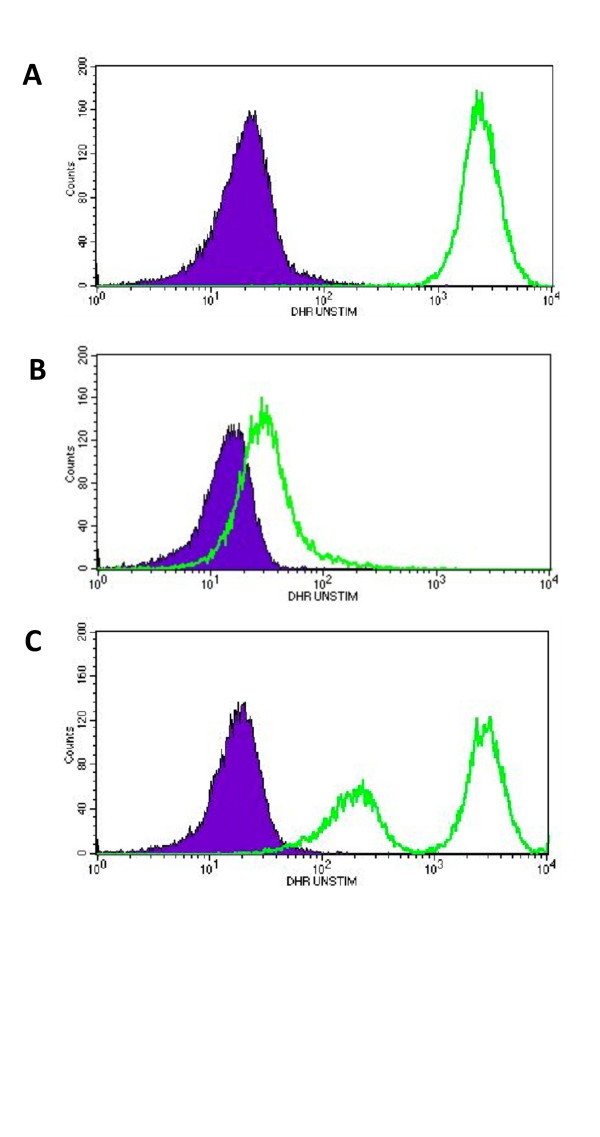
**Dihydrorhodamine (DHR) analysis of oxidation in peripheral blood neutrophils**. In brief, after red blood cell lysis, neutrophils are loaded with DHR. After DHR loading, cells were stimulated for 15 mins with phorbol myristate acetate (PMA) and immediately analyzed by flow cytometry. DHR is a fluorescent dye taken up by neutrophils. When the neutrophils are stimulated and undergo respiratory burst the dye is oxidised with a shift of fluorescence to the right. Shown are (a) normal patient with unimodal shift of fluorescence after PMA stimulation; (b) the patient who has virtually absent shift in stimulated neutrophils consistent with X-linked CGD; (c) the mother of the patient demonstrating dual population of neutrophils in peripheral blood of X-linked CGD carriers.

**Figure 2:**
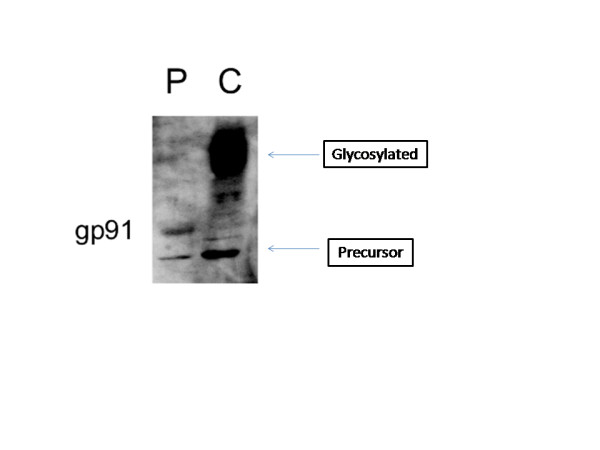
**Western Immunoblot showing gp91^*phox *^protein in the granulocyte fraction of patient (P) compared with a normal control subject (C)**. gp91 expression is significantly decreased with presence of precursor gp91 but absence of glycosylated forms.

In light of the unusual organism, the histology was reviewed and the possibility of chronic granulomatous disease was raised. Neutrophil burst test (Figure 1b) showed impaired neutrophil oxidative burst consistent with CGD. Western blot showed decreased expression of the gp91^phox ^protein consistent with X-linked CGD (Figure 2). His mother and sister were also tested and found to be carriers as evidenced by dual population of normal and abnormal neutrophils (Figure 1c). Genetic analysis confirmed a mutation in exon 9 of the gp91^phox ^gene. The patient was treated with cotrimoxazole and commenced on prophylactic itraconazole. He made a good clinical improvement and was discharged nine days later on prophylactic antibiotics and antifungals. The steroid dose was gradually tapered down. His splenomegaly has gradually improved with the spleen decreasing in size from 24 cm to 18 cm. The haemoglobin and platelet counts returned to normal. His lymphocytes remained low probably reflecting continued enlarged spleen. and after 18 months follow up remains well.

## Conclusion

The production of reactive oxygen metabolites is critical for the elimination of a variety of pathogens including *Staphylococcus aureus, Aspergillus*, and *Burkholderia cepacia complex (Bcc)*. Over the last 20 years bacteria belonging to the Bcc have emerged as an important opportunistic human pathogen. The major clinical interest has focused on pulmonary infections in cystic fibrosis with *Burkholderia cenocepacia *and *Burkholderia multivorans *as accounting for the majority of infections [5]. However, its pathogenicity is not limited to cystic fibrosis patients and CGD patients have been identified as a vulnerable group. *Bcc bacteria are *intrinsically resistant to non-oxidative killing by neutrophils, thus making these patients susceptible to infection. In CGD patients. *Bcc *pneumonia and septicaemia are life threatening and have been associated with fatal outcomes [6]. It is an rare infection in healthy patients, and its isolation should be prompt a search for evidence of immunodeficiency.

In addition to their increased susceptibility to infection, patients with CGD are also prone to a variety of inflammatory and/or rheumatic diseases, which in some cases may be the first clinical manifestations leading to diagnosis [7]. The common inflammatory complications include cutaneous granulomas, inflammatory bowel disease similar to Crohn's disease and a lupus-like syndrome. These inflammatory complications typically respond rapidly to systemic steroid therapy [8]. The finding of granulomas may lead to confusion with other granulomatous conditions including Crohn's disease and, particularly in the presence of a raised serum angiotensin converting enzyme (ACE), sarcoidosis [9,10]. However, serum ACE elevation is associated with activation of the monocyte-macrophage system and may be elevated in a variety of granulomatous and non-granulomatous conditions including ulcerative colitis, tuberculosis and alcoholic liver disease [11]. Although serum ACE is usually increased in sarcoidosis, the test has low disease specificity and needs to be carefully interpreted in the light of the overall differential diagnosis. Hepatic granulomas have previously been reported in adult patients with CGD. In one series, six of seven patients in which liver tissue was available for study had histological evidence of granuloma [12]. In another series 3 of 11 adult CGD patients studied had evidence of granuloma formation on biopsy, one of whom later died from decompensated liver cirrhosis secondary to granulomatosis [13].

Defects in NADPH oxidase are the underlying cause for CGD. The functional NADPH oxidase complex is composed of two membrane bound (gp91^phox ^and gp22^phox^) and two structural cytosolic proteins (gp47^phox ^and gp67^phox^). The proteins gp91^phox ^and gp22^phox ^interact to form a stable heterodimeric cytochrome complex and deficiency of one is usually associated with deficiency of the other protein. The majority of patients are X-linked recessive due to mutations in gp91^phox^, with other forms due to autosomal recessive mutations of the other components each of which is encoded on a different chromosome [8]. Patients with X-linked CGD have a more severe phenotype with earlier presentation, increase in infections and increased incidence of chronic inflammatory manifestations. However some patients with X-linked CGD have a gp91^phox ^protein level ranging from 1% to 25% of normal, so-called X91^-^. These patients often have variable clinical symptoms and may present at an older age [14]. In this case the patients neutrophil burst test showed partial shift suggesting there may be a small residual amount of function. Western blots showed the presence of gp 22^phox ^and gp91^phox ^protein precursor but a decrease in glycosylated forms (Figure 2). Genetic analysis confirmed a missense mutation (937G>A) which has been previously identified as an X91^- ^phenotype with detectable oxidase activity, detectable p22 and slightly detectable gp91 [15]. The patient had done relatively well with no infections until he was immunosuppressed with prednisolone without antibiotic cover; the corticosteroid treatment may have permitted the growth of *B. multivorans*. Corticosteroid treatment may have more severe consequences in these patients and there are been reports in which patients with undiagnosed CGD have been treated with corticosteroids with fatal consequences [16-18].

This case illustrates the importance of having a high clinical suspicion for the diagnosis of immune deficiency in the setting of presumed granulomatous disease and unusual infections. Granulomas have been described in a variety of immunodeficiencies including common variable immunodeficiency, Wiskott-Aldrich syndrome, ataxic telangectasia and severe combined immunodeficiencies [19]. In view of the importance of early diagnosis along with genetic counselling for some families, testing to exclude immunodeficiency should be considered at any age when suspicious symptoms occur, especially in patients with granulomas and atypical or recurrent infections.

## Competing interests

The author(s) declare that they have no competing interests.

## Authors' contributions

AMT drafted the manuscript and finally approved the article for publication. CA has made revisions to the article content. KG provided the Western Immunoblot analysis and reviewed the article. MH has made critical analysis of the article. BH has made revisions to the article content. GPS has provided clinical details, reviewed and revised the article. All authors have read and approved the final manuscript.

## Pre-publication history

The pre-publication history for this paper can be accessed here:



## Figures and Tables

**Table 1:** Liver function and full blood count results

Laboratory Findings	Initial assessment	After 2 days of prednisolone	Admission for splenectomy	On treatment
Albumin (g/l)	29	31	36	44
Bilirubin (μmol/l)	52	25	26	21
Alkaline Phosphatase (IU/l)	2191	1345	966	578
Alanine Transferase (IU/l)	114	89	57	287
Haemoglobin (g/dl)	9.0	8.6	9.8	16.6
White cell count (10^9^/l)	2.2	4.4	1.3	5.0
Neutrophils (10^9^/l)	1.47	3.00	0.76	4.0
Lymphocytes (10^9^/l)	0.43	0.91	0.31	0.51
Platelets (10^9^/l)	223	316	127	162

## References

[B1] Berendes H, Bridges RA, Good RA (1957). A fatal granulomatous disease of childhood: The clinical study of a new syndrome. Minn Med.

[B2] Moltyaner Y, Geerts WH, Chamberlain DW, Heywort PG, Noack D, Rae J, Doyle J, Downey GP (2003). Underlying chronic granulomatous disease in a patient with bronchocentric granulomatosis. Thorax.

[B3] Schapiro BL, Newburger PE, Klempner MS, Dinauer MC (1991). Chronic granulomatous disease presenting in a 69-year-old man. N Engl J Med.

[B4] Lukela M, DeGuzman D, Weinberger S, Saint S (2005). Unfashionably Late. N Engl J Med.

[B5] Cant AJ, Gordon SB, Read RC, Hart CA, Winstanley C (2002). Respiratory infections. J Med Microbiol.

[B6] Lacy DE, Spencer DA, Goldstein A, Weller PH, Darbyshire P (1993). Chronic granulomatous disease presenting in childhood with Pseudomonas cepacia septicaemia. J Infect.

[B7] Winkelstein JA, Marino MC, Johnston RB, Boyle J, Curnutte J, Gallin JI, Malech HL, Holland SM, Ochs H, Quie P, Buckley RH, Foster CB, Chanock SJ, Dickler H (2000). Chronic granulomatous disease. Report on a national registry of 368 patients. Medicine (Baltimore).

[B8] Segal BH, Leto TL, Gallin JI, Malech HL, Holland SM (2000). Genetic, biochemical, and clinical features of chronic granulomatous disease. Medicine (Baltimore).

[B9] Cathebras P, Sauron C, Morel F, Stasia MJ (2001). An unusual case of sarcoidosis. Lancet.

[B10] Ramanuja S, Wolf KM, Sadat MA, Mahoney SJ, Dinauer MC, Nelson RP (2005). Newly diagnosed chronic granulomatous disease in a 53-year-old woman with Crohn disease. Ann Allergy Asthma Immunol.

[B11] Studdy PR, Bird R (1989). Serum angiotensin converting enzyme in sarcoidosis--its value in present clinical practice. Ann Clin Biochem.

[B12] Nakhleh RE, Glock M, Snover DC (1992). Hepatic pathology of chronic granulomatous disease of childhood. Arch Pathol Lab Med.

[B13] Liese JG, Jendrossek V, Jansson A, Petropoulou T, Kloos S, Gahr M, Belohradsky BH (1996). Chronic granulomatous disease in adults. Lancet.

[B14] Jirapongsananuruk O, Malech HL, Kuhns DB, Niemela JE, Brown MR, Anderson-Cohen M, Fleisher TA (2003). Diagnostic paradigm for evaluation of male patients with chronic granulomatous disease, based on the dihydrorhodamine 123 assay. J Allergy Clin Immunol.

[B15] Kaneda M, Sakuraba H, Ohtake A, Nishida A, Kiryu C, Kakinuma K (1999). Missense Mutations in the gp91-phox Gene Encoding Cytochrome b558 in Patients With Cytochrome b Positive and Negative X-Linked Chronic Granulomatous Disease. Blood.

[B16] Trawick D, Kotch A, Matthay R, Homer RJ (1997). Eosinophilic pneumonia as a presentation of occult chronic granulomatous disease. Eur Respir J.

[B17] Kelly JK, Pinto AR, Whitelaw WA, Rorstad OP, Bowen TJ, Matheson DS (1986). Fatal Aspergillus pneumonia in chronic granulomatous disease. Am J Clin Pathol.

[B18] Eppinger TM, Greenberger PA, White DA, Brown AE, Cunningham-Rundles C (1999). Sensitization to Aspergillus species in the congenital neutrophil disorders chronic granulomatous disease and hyper-IgE syndrome. J Allergy Clin Immunol.

[B19] Watters SK, Zacharisen MC, Drolet B, Fink J (2003). Subcutaneous nodules in a patient with recurrent sinopulmonary infections and fatigue. Ann Allergy Asthma Immunol.

